# Current Challenges Facing the Translation of Brain Computer Interfaces from Preclinical Trials to Use in Human Patients

**DOI:** 10.3389/fncel.2015.00497

**Published:** 2016-01-06

**Authors:** Maxwell D. Murphy, David J. Guggenmos, David T. Bundy, Randolph J. Nudo

**Affiliations:** ^1^Bioengineering Graduate Program, University of KansasLawrence, KS, USA; ^2^Department of Rehabilitation Medicine, University of Kansas Medical CenterKansas City, KS, USA; ^3^Landon Center on Aging, University of Kansas Medical CenterKansas City, KS, USA

**Keywords:** brain-computer interface (BCI), microelectrodes, electrocorticography (ECoG), electroencephalography (EEG), closed-loop neuroprosthetic devices, neural prostheses

## Abstract

Current research in brain computer interface (BCI) technology is advancing beyond preclinical studies, with trials beginning in human patients. To date, these trials have been carried out with several different types of recording interfaces. The success of these devices has varied widely, but different factors such as the level of invasiveness, timescale of recorded information, and ability to maintain stable functionality of the device over a long period of time all must be considered in addition to accuracy in decoding intent when assessing the most practical type of device moving forward. Here, we discuss various approaches to BCIs, distinguishing between devices focusing on control of operations extrinsic to the subject (e.g., prosthetic limbs, computer cursors) and those focusing on control of operations intrinsic to the brain (e.g., using stimulation or external feedback), including closed-loop or adaptive devices. In this discussion, we consider the current challenges facing the translation of various types of BCI technology to eventual human application.

## Introduction

Brain-computer interfaces (BCIs) and their applications for treatment of nervous system damage have shown enormous progress as functional restoration tools in pre-clinical studies. In general, most BCIs are designed to bypass damaged structures and fiber tracts. BCIs range from common devices, such as cochlear implants that use externally recorded sound to directly stimulate auditory nerve fibers, to devices that derive control signals from cortical activity, allowing individuals with paresis to operate a prosthetic device. Other BCIs are designed to aid in acute rehabilitation training sessions. Regardless of the type, the major purpose of BCIs is to improve the quality of life for the patients who use them.

Damage to the nervous system can result in profound sensory, motor, and cognitive deficits that strongly impact day-to-day functioning of afflicted individuals. The type and extent of these deficits are dependent upon the location and extent of the injury. Injuries affecting motor cortex, such as might occur after a focal traumatic brain injury or stroke, can lead to impaired use of digits, limbs, or whole regions of the body due to loss of descending corticospinal neurons or disruption of sensory-motor integration. Spinal cord injury impacts communication of neural signals at the site of injury, leading to motor, sensory and autonomic deficits. For these types of injuries, there are no effective post-acute restorative treatments. Research in stem cell therapy to regenerate damaged neurons that could restore damaged pathways is currently underway (Gavins and Smith, 2015; Hosseini et al., 2015; Sharma et al., 2015; Sullivan et al., 2015), but is likely years from fruition. Recovery after central nervous system (CNS) injury is thought to manifest itself through neuroplastic mechanisms, which have been shown to be aided through rehabilitative therapy (Nudo et al., 1996; Nudo and Friel, 1999). Dramatic recovery from motor deficits has occurred in some cases (Bajaj et al., 2015; Warnecke et al., 2015), but recovery from neurological injuries rarely results in a full restitution of function. Effectiveness of any therapy is constrained by the type and extent of injury, efficiency of neuroplastic mechanisms involved, and type of intervention. BCIs offer a pathway, in conjunction with rehabilitative therapy, for promoting restitution of function.

Current technology available for clinical populations ranges from simple devices that stabilize a shaking hand (Popović Maneski et al., 2011; Grimaldi et al., 2013), to devices that augment the ability of a patient with locked-in syndrome to communicate with others (Holz et al., 2015). While these technologies offer promise for recovery from or for relief of symptoms of CNS injury, there are still many challenges in the integration of BCIs into effective prosthetic devices. These challenges include adequate spatiotemporal resolution in interpreting information recorded from the brain for naturalistic control, decoding a sufficient number of degrees of freedom to maintain natural movements, integration of feedback mechanisms, easing the technological support needed for integration of the BCI and reducing the invasiveness of components while maintaining the longevity of signal acquisition. Additionally, a number of recent studies have focused on devices contained entirely within the CNS that create artificial links between related areas. Here, we focus on the advantages and disadvantages of various approaches to interfacing BCI devices with the nervous system, based on results from both pre-clinical and clinical studies. We highlight the challenges associated with the implementation of high fidelity BCI devices to a clinical setting, possible methods for overcoming these challenges, and the distinction between devices that control extrinsic operations and those that control operations intrinsic to the CNS.

## BCI Operating Modes

When considering potential clinical interventions using neural prostheses, a convenient way of classifying devices is based on whether they control extrinsic or intrinsic operations. In this review, BCI devices that operate primarily by detection of electrical signals from the CNS are mainly considered, as techniques for recording other measures of CNS information (i.e., magnetic, metabolic) are typically unwieldy for chronic use or cost prohibitive.

### Control of Extrinsic Operations

Neural prostheses are classified as controlling extrinsic operations when the device contains a decoder that records CNS signals in real-time, modifies those signals via a control algorithm and outputs the translated and modified signal to a body-external device such as a prosthetic or robotic limb or a computer cursor. In this way the individual gains control over an artificial device that has the possibility to be incorporated into the body schema.

**Figure 1:**
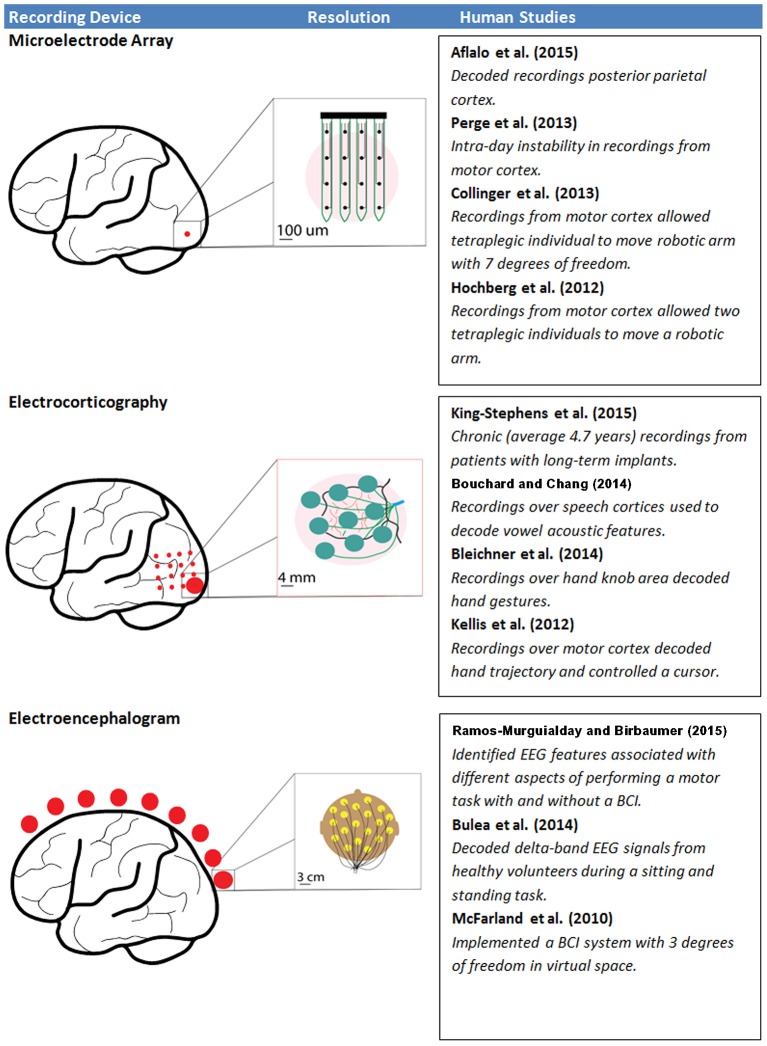
**The resolution of each type of recording interface, as well as a selection of recent human studies associated with each interface.** Red dots represent the relative extent of recording interface placement, while inserts demonstrate the scale and possible arrangement of electrodes at that site.

A limitation of devices controlling extrinsic operations is that accuracy in decoding movement intention is typically gained through an increase in the number of recording channels (Carmena et al., 2003); however, increasing recording channels brings the challenge of increasing channel density in a particular location of interest. Depending on the type of information being recorded and the decoding strategy, the increase in computational burden and power required from adding greater numbers of channels may also become nontrivial. Likewise, increasing the invasiveness of the electrodes can lead to increases in decoding accuracy, but at the cost of increased surgical risk and potential immune response (Ward et al., 2009). Additionally, chronic recordings are prone to drift in intent decoding, making repeated calibration necessary. Although these limitations prevent the widespread use of these BCI systems in clinical settings, studies to date are encouraging and represent tangible evidence of the type of functional restoration that can be achieved using BCIs. Here, BCIs controlling extrinsic operations are classified into three different categories based on the electrode interface used for signal acquisition from the CNS (Figure 1). These include two invasive electrode-CNS interface approaches [microelectrode array (MEA) recording, electrocorticography (ECoG)] and one non-invasive electrode-CNS interface (electroencephalography, EEG).

#### Microelectrode Array Recording

MEA recording, used in animal models for decades, represents the most invasive BCI approach, as penetrating microelectrodes are placed within the brain structure itself, typically within the gray matter of cerebral cortex. Though the technology was initially developed in animal models, a relatively small number of human studies have now been conducted with implanted MEAs. Microelectrode probes can range from a single-shank electrode to arrays consisting of tens of thousands of recording sites. The specific pattern and distribution of sites allows for dense population recordings throughout a single or multiple regions of interest. MEAs allow the highest spatial and temporal resolution of any type of neurophysiological recording system used in BCIs (Obien et al., 2015), but at the expense of spatial coverage at the site of recording. The use of MEAs allows for detection of the extracellular electric field changes reflecting the membrane potential of the individual neurons closest to the tip of each microelectrode.

While the voltage changes are quite small, neuronal action potentials, or spikes, can be detected within the electrical signal since rapid changes in membrane potential associated with the opening and closing of membrane ion channels have a characteristic temporal pattern. Due to their rapid onset and offset, the resulting detected spikes can be effectively reduced to point processes using voltage thresholding, simplifying the design of decoding algorithms, especially when large MEAs are employed. Further analysis using automated or semi-automated clustering algorithms or manual feature detection allows classification of multiple individual neurons recorded from a given recording site, increasing the accuracy of decoding (Todorova et al., 2014). It should, however, be noted that the process of detecting spikes introduces another source of error, with some techniques sacrificing accuracy for the sake of computational expedience (Rey et al., 2015). Depending on the information that needs to be obtained from spike trains, these errors can have a nontrivial significance (Pazienti and Grün, 2006).

Once spikes have been detected and multiple neuronal spikes discriminated (if desired), typically the rate of firing (i.e., spike rate) of the individual neuronal components is calculated. Both accuracy and ease of computational processing are dependent upon the combined choice of a spike rate estimator and a spike rate decoder, with simpler methods allowing computations to be performed on a millisecond time scale and more complex, probabilistic models limiting computations to seconds or even minutes (Cunningham et al., 2009). Based on these temporal limitations, the practical need for real-time adaptation when implementing a BCI makes some of the simpler methods more attractive (Cunningham et al., 2011). To this end, it has been demonstrated that the use of a closed-loop, adaptive decoder can also lead to increased simultaneous neural adaption, resulting in improved skill retention (Orsborn et al., 2014).

Preclinical BCI research in animal models has typically utilized implanted MEAs chronically embedded in the cortex for decoding movement intention. The rationale for this approach dates to the 1960s when Evarts found that neurons in the motor cortex of non-human primates (NHPs) altered their firing patterns immediately prior to the onset of movement (Evarts, 1966) and was later strengthened when Fetz (1969) demonstrated that neuronal firing rate could be volitionally controlled. More recent studies in NHPs demonstrate a consistent ability to decode signals to move transiently paralyzed limbs (Ethier et al., 2012), a simulated or robotic limb (Wessberg et al., 2000; Carmena et al., 2003; Velliste et al., 2008; Willett et al., 2013), or a cursor on a screen (Taylor et al., 2002; Wu et al., 2004; Nuyujukian et al., 2014), and even predict hand orientation with extremely high accuracy (Peng et al., 2014).

MEAs have proven resilient in producing reliable signals from a single area over periods of up to a year (Flint et al., 2013). However, longevity of single unit recordings with indwelling electrodes has been one of the major limitations of this approach. For example, studies have shown a decay in signal strength over the course of 100 days (Rousche and Normann, 1999), and large performance variability between trials and type of electrode used (Ward et al., 2009). Furthermore, information generated by decoders has been shown to diminish over extended implantations (Nuyujukian et al., 2014). This somewhat variable, and arguably short, lifetime limit for recording robust signals from implanted MEAs still needs to be addressed by future improvements in MEA materials technology. While estimates of the number of neurons needed to decode arm movements off-line range between 150 neurons with serial single unit recordings and 600 units from MEAs (Georgopoulos et al., 1986), accurate on-line BCI control is possible with far fewer recorded units due to the closed-loop adaptation that occurs when learning BCI skills (Taylor et al., 2002; Carmena et al., 2003). This phenomenon could be a key to improving long-term patency of indwelling MEAs. If it is possible to use only a subset of sites to generate information for decoding, then as those sites slowly lose functionality it may be possible to use redundant sites, allowing for an extended prosthetic lifespan.

Although MEA studies in humans are limited due to their invasive nature, recent results indicate the advantages of using such high-resolution paradigms. Aflalo et al. (2015) found that the decoding of spike trains associated with motor imagery in a patient chronically implanted with MEAs embedded in the posterior parietal cortex resulted in the smooth movement of a robotic limb with 17 degrees-of-freedom. Two 96-channel MEAs were embedded for 21 months with no signs of adverse effects. The subject was asked to imagine reaching toward a specific goal, and channels that demonstrated preferential firing when the subject imagined achieving the goal were discriminated from neurons tuned to trajectory. When these goal-tuned units were used as tuners for accomplishing a specific task, decoding accuracy was higher for a given number of units. It should be noted, however, that the goal-tuned units changed over time, indicating that an adaptive decoder would be important for this sort of prosthetic device to be implemented in the future for long-term implantations. This problem of varying tuning is also seen in recordings from units in motor cortex (Perge et al., 2013).

These changes in tuning were most likely due to physiological changes in the neuronal firing patterns as a result of adaptation to the decoder. As the patient learns to operate the BCI, functional reorganization occurs in multiple brain areas, resulting from closed-loop feedback and adaptation to performing the new BCI-related task, and presumably allowing a smaller number of units to function in tuning the device (Taylor et al., 2002; Carmena et al., 2003). The ability to produce a smooth movement based on the decoding of a goal-tuned unit represents a significant divergence from previous studies involving chronically implanted MEAs in human patients. These studies used motor cortex (Hochberg et al., 2006, 2012; Collinger et al., 2013) as an area for control, and were quite successful; however, a noted limitation was that movements produced using these systems were slower and somewhat inflexible (Hochberg et al., 2012) when compared to natural reaching movements. Thus, the ability to use a goal-tuned unit in posterior parietal cortex as a control source for decoding intent using motor imagery could serve as an informative alternative to decoders focused on motor cortex.

There are several additional challenges related to using MEAs in BCIs for clinical populations. The insertion of MEAs into cortical tissue is an invasive procedure requiring a craniotomy and resection of the dura. The surgical procedure introduces a possible pathway for infection. MEA implantation can lead to small-scale tissue damage that increases with a greater number of implants. Glial scarring occurs at the insertion site, and is thought to be a major factor reducing the longevity of useable signals that can be recorded in a chronically implanted individual. Another major problem of chronically implanted MEAs is micro-motion, which causes the formation of scar tissue, leading to a decrease in the quality of recordings over time (Williams et al., 2007; Ersen et al., 2015). Current materials research is focusing on changing various properties affecting the stiffness of the microelectrode, in the hope that scar tissue formation caused by micro-motion will be minimized (De Faveri et al., 2014). Obien et al. (2015) provide a comprehensive review of the different types of MEAs currently in use. The viability of MEAs in clinical use may ultimately depend upon further advances in materials research (McCarthy et al., 2011; Tooker et al., 2012; Felix et al., 2013; De Faveri et al., 2014).

**Figure 2:**
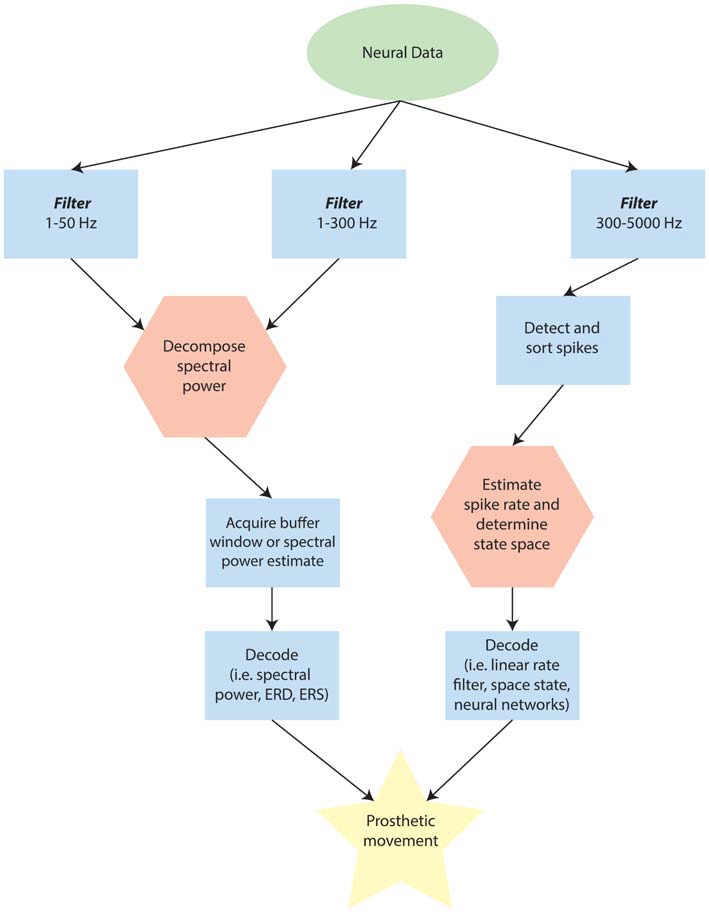
**Schematic of possible differences in analysing point-processes and waveforms when using externally interfaced motor prosthetics.** Note that this flow may change depending on the specific device, but is designed to provide a broad overview for comparison. In the diagram, the green ellipse represents data that has been recorded and amplified from the neural source. Blue rectangles are stages along the processing pathway that are typical for many devices. Red hexagons represent potential rate-limiting steps in determining the latency of the Brain Computer Interface (BCI) response to immediate internal changes in patterns of neural activity.

A potential solution for MEA signal stability would be to implement BCIs that utilize somewhat lower-fidelity neural signals. One signal that can be acquired by MEAs is the local field potential (LFP). The LFP represents the summation of active cellular processes nearest the site of each microelectrode. While the general process of analysing LFPs is similar to analysing spike data, the computational stage and filtering is somewhat different (Figure 2), and some delay is inherent due to the latency with which changes in spectral power occur and can be measured. Despite these limitations of LFPs, the advantages of increased recording stability may outweigh the loss in accuracy.

A combination of lower-fidelity LFP recordings and spike recordings might also be desirable. It is possible to generate predictions of the imagined single-joint movements in a tetraplegic individual by decoding the joint trajectory using the LFP frequency signals and multi-unit spike activity similarly to those predicted by decoding single-unit activity (Ajiboye et al., 2012). Recent work by Hall et al. (2014) indicates that it is possible to estimate single unit firing rates using the slow potentials from LFPs derived at several cortical locations. If this method can be applied to estimate the single unit firing rate of a single unit, without the need for first gathering spike data to calibrate the estimation, it could lead to the development of a BCI with sufficient longevity that still offers good spatiotemporal resolution. However, it is important to note that the filters used to perform the necessary calculations to deconvolve the firing rate of a single neuron from the low frequency LFP signals using current methods require prior knowledge of spike train information from multiple neurons. Furthermore, when using single-unit activity decoded from LFPs, there is an additional step of transforming the data during which accuracy could be lost. Despite these limitations, the method described by Hall et al. (2014) offers the added benefit of allowing accurate single unit firing rate predictions over the course of several weeks, which is an improvement on intra-day instabilities in decoding from single-unit activity itself (Perge et al., 2013). In this way, using LFP decoders in conjunction with single- and multi-unit activity may be a key step in implementing long-term implants.

#### Electrocorticography Grids

ECoG consists of a mesh or grid of electrodes distributed across the cortical surface that can be placed either subdurally or epidurally. This technique can detect the LFPs from the cortical surface at specific locations, but does not have the resolution to detect individual spikes. As less invasive interface approaches are used, the focality of the recorded signal necessarily is degraded. What ECoG lacks in spatiotemporal precision with respect to individual spiking profiles, it makes up for in patency. ECoG has shown resilience in long-term recordings in human patients implanted for up to 7.1 years (King-Stephens et al., 2015). In addition, ECoG has the ability to expand the extent of spatial coverage relative to MEAs. For example, signals can be detected and decoded simultaneously from M1, PMd, and S1. Additionally, using this method, it is possible to detect not only recordings from gyrus, but also from the sulcal wall (Yanagisawa et al., 2009), albeit via a more invasive process.

In humans, most studies investigating the use of ECoG for BCI applications have used clinical electrodes implanted in epilepsy patients for localization of epileptic foci with an electrode size on the order of a few millimeters and an interelectrode distance of approximately 1 cm. In particular, movement-related spectral power changes have been shown to occur not only during overt movements of skeletal musculature, but also during imagined movements (Leuthardt et al., 2004), indicating that these spectral power changes may be useful in motor-impaired patients who are unable to perform overt movements. Furthermore, several studies have demonstrated that functionally motor-intact human patients can modulate the spectral power of ECoG signals to achieve on-line control of a computer cursor (Leuthardt et al., 2004; Wilson et al., 2006; Felton et al., 2007; Schalk et al., 2008).

ECoG has also been used to implement BCI devices in motor-impaired patients. A study in a hemiparetic patient demonstrated that it is possible to use ECoG to control a prosthetic arm using recordings from sensorimotor cortex (Yanagisawa et al., 2011). Additionally, the use of ECoG signals for control of a BCI system with three degrees-of-freedom based upon motor imagery of movements at multiple independent joints has been demonstrated in a quadriplegic patient with good signal quality for durations up to 1 month (Wang et al., 2013). While on-line BCI control in human patients with ECoG has been limited to short durations, with relatively large electrode sizes, arrays with sub-millimeter electrode sizes have been proposed as a means to obtain signals with increased spatial specificity. These micro-scale arrays have been utilized for online BCI control experiments in NHPs (Leuthardt et al., 2009; Rouse et al., 2013). Importantly, these studies utilized chronic epidural recordings, demonstrating the stability of ECoG signals as well as the potential to implant ECoG BCI systems on the surface of the dura, which would reduce the risks of infection due to isolating the implant from the subdural space.

While closed-loop BCI systems generally have used changes in spectral power associated with imagined movements of a single joint in humans or high gamma power in arbitrary electrodes in NHPs, a more natural control algorithm may be to use signals decoded from natural movements or behaviors. The potential for this type of BCI using ECoG has been demonstrated by studies that have used ECoG signals to decode 2D movement directions in rats (Slutzky et al., 2011) and NHPs (Flint et al., 2012) and to continuously decode movement kinematics of 2D (Flint et al., 2012; Marathe and Taylor, 2013) and 3D arm movements in NHPs (Chao et al., 2010). Along with animal models, ECoG recordings from human epilepsy patients have been used to decode information about voluntary movements. ECoG recordings have been used to classify movement directions of arm and hand movements (Reddy et al., 2009; Wang et al., 2012; Chestek et al., 2013). Similarly, it is possible to decode continuous finger flexion/extension (Chestek et al., 2013) and 2D arm and hand trajectories using ECoG with modest accuracy (Schalk et al., 2007; Pistohl et al., 2008; Sanchez et al., 2008; Kellis et al., 2012), as well as move a cursor to an onscreen target using full neural control with no trajectory decoding (Kellis et al., 2012). Flint et al. (2014) extended these findings to show that it is possible not only to determine trajectory, but kinetics for use in functional electrical stimulation as well using ECoG (Flint et al., 2014). There are also preliminary indications that ECoG in patients with stroke and epilepsy can be used to predict three degrees-of-freedom in arm trajectory during motor imagery (Nakanishi et al., 2013). Other recent experiments have used high-density ECoG placed over specific areas to yield high accuracy decoding. Placement over the speech cortices yielded accurate prediction of vowel acoustics during speech (Bouchard and Chang, 2014), and placement over the hand knob area in sensorimotor cortex resulted in decoding of hand gesturing (Bleichner et al., 2014), with high frequency signals (>65 Hz) showing the most accurate results. In general, it should be noted that the higher frequency signals tend to produce more accurate results, presumably in part because there is a shorter latency between intent and decoding/feedback.

#### Electroencephalography Caps

EEG is the least invasive technique, but also provides signals with the broadest spatiotemporal coverage of the cortex. Similar to ECoG, EEG detects general electric fields that are a sum of the electrical activity for a given region. However, as the EEG signal detection is somewhat distant to the site of interest (e.g., the precentral gyrus) there is an inherent limitation to spatial and spectral resolution during signal acquisition. Because the voltage from a dipole falls off with the inverse of the square of the distance from the dipole, the extra distance between neural sources in the cortex and EEG electrodes causes a summation over a wider range of cortex (Cooper et al., 1965). The spectral resolution limitation is due primarily to the fact that higher frequency signals, which are more focal, tend to be averaged out by the low spatial resolution. In addition, high frequency activity in general is lower in amplitude than low frequency activity, and can be filtered out from the inherent dampening of the bone and tissue that it must travel through Cooper et al. (1965) and Pfurtscheller and Cooper (1975). Finally, EEG signals are also susceptible to contamination from electromyographical (EMG) artifacts or eye blinks (Cooper et al., 1965; Wolpaw and McFarland, 2004). Due to these complications, trajectory predictions using EEG are generally not as accurate as those using MEA recording or ECoG.

Despite these limitations, EEG provides an excellent method for obtaining neural information from patients in a clinical setting without the need for surgery. EEG is also promising for use in acute settings that could be associated with rehabilitation and behavioral recovery, since it is non-invasive in nature. One of the hopes for EEG is that by using proper placement of a sufficient number of leads and a significant amount of prior data in healthy patients, it will be possible to use frequency signatures from different areas to overcome some of the spatiotemporal problems listed previously. As noted previously, EEG has the advantage of broad spatial coverage in recordings. It may be possible to turn this broad spatial coverage into an advantage in resolving the origin of activity in the brain. There is a large body of work in EEG source imaging that focuses on estimating the location of current sources for scalp measurements by solving the so-called static electromagnetic inverse problem. This is done using the collection of scalp measurements as well as a set of reasonable* a priori* constraints based on the assumed or measured physiology of the brain to determine the most likely origin of the current source or sources. As Michel et al. (2004) detail in their review of such techniques, such estimates depend on a number of factors, including but not limited to the number and position of electrodes on the scalp, the solution algorithm used to solve the inverse problem, and the integration of MRI data to serve as a prior. Recent studies demonstrate that it is possible to incorporate such source estimation techniques to EEG recordings for potential use in future BCI applications (Aihara et al., 2012; Yoshimura et al., 2012).

Although a variety of signal analyses have been used for EEG BCI systems (Blankertz et al., 2004), a more traditional approach has been to utilize average features of the frequency spectrum in relation to a motor event. A common strategy is to identify periods of event-related desynchronization (ERD) as a cue for some BCI output. ERD itself is a decrease in a pre-defined spectral frequency band that can have a different physiological interpretation depending on the context of the task. Controlling a BCI system with ERD associated with motor movements has particular relevance to motor-impaired populations. Because ERD has been shown to occur with imagined in addition to overt movements, it is applicable as a BCI control signal in patient populations that are unable to execute motor actions (Pfurtscheller et al., 1997). The application of EEG ERD-based BCI systems has been demonstrated in normal controls and patient populations (Wolpaw et al., 1991; Pfurtscheller et al., 2003; Blankertz et al., 2004; Wolpaw and McFarland, 2004; McFarland et al., 2010). While EEG is a powerful tool due to its ease of use and non-invasiveness, its use in BCI system development is hampered by the limitations described above. To date the best performance of an EEG BCI system in control of extrinsic operations is three degrees-of-freedom, which was only achieved after months of intensive training (McFarland et al., 2010).

Although EEG-based BCI that use ERD and event related synchronization (ERS) in various frequency bands are common, recent work has aimed at providing a more comprehensive picture of changes through various power bands through the duration of a variety of tasks. Depending on the task, and thereby, the neural circuits involved, different signal features may be important at different times relative to the event of interest. A recent study identified EEG features in healthy subjects related to several stages of motor activities (Ramos-Murguialday and Birbaumer, 2015). Ideally, when using EEG to control a BCI, the different components of a movement would have distinct feature signatures that could be detected. Indeed, in this study it was noted that there were distinct features during active and passive proprioception, active intention, and passive involvement in motor activity. Importantly, these features were significantly different when performing a BCI task as compared to other motor tasks, indicating that decoder design must take into account changes in EEG features depending on the type of activity involved.

Other less time-sensitive applications than fine motor movement may lend themselves to BCIs that utilize even lower frequency signals, sometimes referred to as slow cortical potentials (SCPs) or movement-related cortical potentials (MRPs). In these cases accuracy can be added by including pre-processing steps using a variety of methods to reject false positive signals. A recent study has demonstrated that it may even be possible to decode movement intent from delta-band (0.1–4 Hz) features, showing high accuracy in movement classification during a sitting-to-standing task in healthy volunteers (Bulea et al., 2014). In fact, BCIs using slow signals have application even beyond motor tasks, such as allowing communication via a spelling device for patients with locked-in syndrome (Birbaumer et al., 1999) or even allowing web-browsing for paralyzed patients (Bensch et al., 2007). Another recent direction for improving accuracy is seen in the development of the brain/neuronal computer interface (BNCI). The recent distinction between BNCI and BCI devices draws on the fact that the BNCI makes use of other signals or current sources recorded from the body that are not located directly in the brain. Soekadar et al. (2015b) demonstrated that it is possible to use electrooculography (EOG) in conjunction with EEG to improve use of a grasping hand exoskeleton.

### Control of Intrinsic Operations

Some implantable devices operate by modifying the flow of information or causing modifications in the functional neural networks of the brain. These devices control what can be considered intrinsic operations in the brain. Devices in this category fall into two sets: open-loop and closed-loop stimulators. In open-loop stimulation, some form of stimulation is applied to a region of the brain with a frequency that is often determined using physiological parameters, but not necessarily correlated to the immediate activity of the brain. Such open-loop devices deliver a constant stream of current to the site of interest, as is predominant in deep brain stimulation (DBS), although recent studies have used closed-loop DBS for treatment of Parkinson’s Disease (PD), epilepsy, and intention tremors, as will be noted. There is also interest in the application of open-loop stimulation in conjunction with BCI therapy; however, in the scope of this review we will mainly cover closed-loop devices.

#### Closed-Loop Controlled Intrinsic Operations

**Figure 3:**
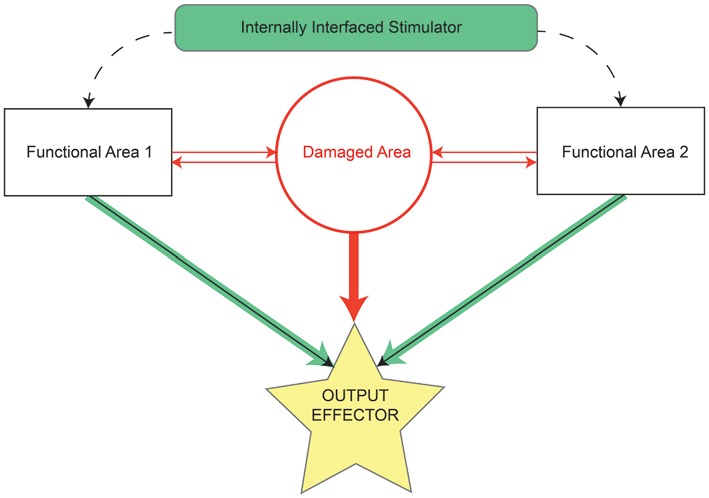
**Schematic by which internally contained stimulation devices restore lost function resulting from damaged or missing tissue.** Before damage, the area of interest (red circle) and functionally related areas (rectangles) relay information between each other and effectors (solid arrows) of some output task (yellow star). The majority of information in controlling task output initially comes from the damaged area (thick red arrow), but may also arrive, although to a lesser extent, from functionally related areas (thin black arrows). Following injury, connections to and from the damaged area are lost (all red elements). The stimulation device serves as a direct bridge between functional areas, allowing strengthened output (thick green arrows) from those areas to the output effectors and thereby restoring some degree of lost functionality.

By nature of their application in primarily remedying some sort of functional deficit to patients, most BCIs could be broadly considered as closed-loop devices by virtue of the feedback a patient receives, typically visually, from the device when using it. In this review, we will be more precise with the definition of closed-loop strategies, and break “closed-loop” into two subcategories. The first subcategory of closed-loop strategies incorporates the ongoing activity patterns in individual neurons or ensembles of neurons to determine when an electrical stimulus will be applied in another location. Thus, intrinsic control can facilitate the flow of information from one region of the brain to another (Figure 3). These strategies are not driven by patient recognition of some extrinsic goal, but rather form a completely internal closed-loop. Feedback from an applied stimulus that controls intrinsic operations is typically less overt, as electrical stimulation is generally at subthreshold levels for generating sensation or movement, and measures of functional outcome are harder to ascertain on a trial-by-trial basis. In contrast, such feedback results in gradual changes in network connectivity, cognitive function or memory. This feature of intrinsic control provides an additional challenge since the network changes are thought to rely on Hebbian learning mechanisms, as are discussed below. While it is true that at the synapse such a circuit would comprise a feed-forward system, it is generally the case that reciprocal anatomical projections exist between cortical areas (Donoghue and Parham, 1983; Zhang and Deschenes, 1998), leading to bi-directional information flow and thus closing the loop. Stimulation in this paradigm requires not only a high-fidelity signal to detect and decode trigger events, but a highly focal, transient stimulus delivery. This is relatively simple when using MEAs to deliver the stimulus, but becomes more challenging when using less focal types of stimulation such as epidural stimulators or transcranial magnetic stimulation that stimulate relatively large volumes of tissue.

In PD, it is thought that DBS can improve motor functioning by disrupting abnormal activity. To improve upon existing, open-loop DBS methods, one study in eight PD patients used frequency characteristics of LFPs recorded from the subthalamic nucleus (STN) to determine when to stimulate. Since beta frequencies (13–30 Hz) are thought to correlate with impairment in PD, stimulating the STN only during periods of high beta activity provides an adaptive, or closed-loop, approach to DBS (Little et al., 2013). This adaptive DBS caused a significant increase in subjects’ neurological scores compared with continuous or random DBS. A closed-loop BCI to control intrinsic operations has also been used in epilepsy patients. In a randomized multicenter double-blinded controlled trial of 191 subjects, ECoG electrodes were used to detect epileptiform activity in the recorded signal (Heck et al., 2014). Following detection of epileptiform activity, brief pulses of electrical stimulation were applied to the seizure focus, an approach known as responsive focal cortical stimulation (RNS). Subjects receiving RNS showed a significant reduction in partial-onset seizures after 2 years in the study. In treatment of intention tremors, surface electrodes recording EMG activity have been used to create a sort of closed-loop on-demand control system for DBS that may reduce patient resistance to treatment by stimulation (Yamamoto et al., 2013).

Aborting pathological activity using feedback-controlled electrical stimulation is just one application for closed-loop control of intrinsic operations. Another application is to facilitate synaptic efficacy of specific neural connections, using the natural timing of neuronal firing between groups of neurons. This approach derives its rationale from Hebbian plasticity theory, which posits that neuronal connections are strengthened when presynaptic activity is temporally linked with post-synaptic activity. Because synaptic efficacy changes rely on precise millisecond by millisecond timing relationships, approaches to investigate closed-loop control in this context necessarily require the highest temporal resolution possible. To date, this has been achieved only with MEAs recording individual neuronal spikes. Jackson et al. (2006) showed that it is possible to modulate activity of neurons within the motor cortex based on a spike-dependent stimulation paradigm. In this model, monkeys with chronically implanted microelectrodes in two nearby populations of neurons in the motor cortex were trained on a torque-tracking task. The two populations were tuned to different trajectories. However, when one of the microelectrodes was stimulated based on the spikes recorded from the other microelectrode, the trajectory tuning became similar between the two populations. This study suggested that it is possible to alter existing cortical connections by “linking” two areas together using closed-loop stimulation. Of added interest is the fact that these changes persisted even after the closed-loop period ended, indicating that it was possible to induce long-term changes in synaptic efficacy using this paradigm.

Extending this idea to a traumatic brain injury model, Guggenmos et al. (2013) showed that it is possible to restore a reaching function in rats following damage to motor cortex by linking the premotor and somatosensory areas using activity-dependent stimulation (ADS; Guggenmos et al., 2013). In this study, a focal impact was made over the rat’s caudal forelimb area in motor cortex, abolishing its ability to perform the reaching task effectively, largely due to the disruption in somatosensory motor integration. A recording microelectrode was implanted in the spared rostral forelimb area (RFA), which is somewhat analogous to the primate premotor area. A stimulating electrode, which was triggered by a wireless, battery-operated, head-mounted chip, was implanted in the primary somatosensory (S1) forelimb area. In the ADS paradigm, which ran continuously 24 h a day for up to 28 days, spikes detected in RFA were used to trigger stimulation in S1 after a brief delay (7.5 ms). Remarkably, rats in the ADS protocol demonstrated a significant recovery of functional reaching behavior within a few weeks of ADS treatment. In conjunction with behavioral improvement, synaptic potentiation between S1 and RFA increased as well. Recently, a version of this paradigm has been applied to the rat cervical spinal cord as well, demonstrating a possible treatment mechanism for spinal cord injury, although the trigger signal was EMG activity and not primary CNS activity (McPherson et al., 2015).

There are still a number of unanswered questions regarding the effects of ADS in the context of neuronal pathologies. For example, it is not yet known how long the effects last, or the duration of the therapeutic window. Nevertheless, such a strategy of changing synaptic efficacy is an attractive option for use in a temporary implant because it raises the possibility of a removable or degradable device that only needs to function transiently. Thus, the simplicity of use of the device would mean a one-time surgical operation for patients, with the possibility of having a degradable or removable device that could then either be left *in situ* or explanted after treatment. In addition, aside from setting the thresholds for spike detection, there are a minimum of decoding algorithms that must be customized for individual patients, increasing the feasibility of such an approach in a clinical setting.

Other devices that control intrinsic operations have utilized a different approach. These devices restore cognitive function by replacing circuitry of the brain that is missing or malfunctioning (Berger et al., 2011, 2012; Hampson et al., 2012; Opris et al., 2012; Bonifazi et al., 2013). Berger’s group demonstrated that it is possible to improve rat memory scores in a delayed non-match-to-sample task by implanting a device to translate spike trains detected in CA3 into stimulus trains in CA1 (Berger et al., 2012). Presumably, this closed-loop stimulation acts as a proxy for lost hippocampal function, modifying the spatiotemporal coding of the neural spike information in a similar way to the intact brain.

A major remaining challenge for these types of devices is that in order to increase the degree of complexity of information transmitted, it is necessary to increase the number of inputs. This problem presents a similar challenge as in the externally operating device case in that there is a density limit to the number of electrode sites that can record from a given area at a particular time. As the number of inputs increases, the computational difficulty increases as well. Put in context, a 2004 study by Izhikevich that modeled 100,000 neurons with 8.5 million connections between them took roughly 60 s of computation time for every 1 s of simulation time (Izhikevich et al., 2004). While technology has improved substantially since 2004, it is easy to imagine that as the number of neurons increases the computational difficulty will increase quickly, too. Thus, the complexity of the cognitive task being recovered will most likely determine the feasibility of employing such techniques.

As mentioned previously, a second strategy exists when incorporating closed-loop strategies for control of intrinsic operations. These types of devices are commonly classified as “restorative” BCI, as they are primarily used in rehabilitation treatments as a means to train patients to overcome some form of impairment. While they technically do affect some element extrinsic to the patient, the goal is to cause lasting intrinsic plastic changes that remedy deficits and eventually allow the patient to no longer need the use of the BCI; thus they are classified here with the other intrinsic devices. For example, a BCI designed to reward desynchronization of particular oscillatory rhythms in stroke patients with corresponding proprioceptive feedback by movement of an orthosis demonstrated a clinically meaningful change in assessment scores of patients receiving the orthotic treatment against controls (Ramos-Murguialday et al., 2013). This type of training BCI has been the subject of much interest in the field. One direction is the adjunctive use of non-invasive electrical stimulation with training BCI to enhance learning by amplifying the ERD signal using anodal transcranial direct current stimulation (Soekadar et al., 2014; Kasashima-Shindo et al., 2015; Soekadar et al., 2015a). Another avenue is the use of graded velocity feedback in response to the relative strength of the ERD signal to improve learning by providing improved visual and proprioceptive feedback during BCI-triggered orthotic movement (Soekadar et al., 2011). In this study, even stroke patients demonstrated improved modulation of ipsilesional activity; a similar study demonstrated evidence that this paradigm could lead to new voluntary EMG activity in hemiparetic patients (Shindo et al., 2011). There are a few case studies involving BCI for modulating intrinsic operations as well. One study used visual feedback for the control of excessive levels of beta band activity detected by EEG, providing some evidence that this paradigm could cause voluntary changes in pathological brain activity and improve handwriting for a patient suffering chronic writer’s cramps (Hashimoto et al., 2014). A within-subject withdrawal design in functional EEG BCI-driven neuromuscular electrical stimulation showed some restoration of voluntary EMG activity in a paretic patient where previous rehabilitation treatments had failed (Mukaino et al., 2014). Methods of non-invasive stimulation that could tentatively be used with some of the aforementioned strategies have been proposed, but are still in preliminary stages (Soekadar et al., 2013; Wilde et al., 2015; Zrenner et al., 2015).

#### Evaluating Intrinsic Operation Efficacy

While many of the restorative closed-loop BCI devices have demonstrated substantial clinical efficacy, one common theme among closed-loop devices that do not fall under this category is that they are still farther from clinical application when compared to their externally interfaced counterparts. This may be, in part, because the underlying mechanisms of some of the internally interfaced devices are still not well understood. For example, in a device attempting to recreate the firing patterns connecting one region to another, what sort of simulated pattern would be important to use? Or in the stimulation-dependent closed-loop system, how does the “linkage” between the two areas occur? Before the translation of these devices to a clinical setting, there remains a large amount of investigation to understand the mechanistic means by which these devices work. Even in those devices that are closer to widespread clinical implementation, the neuronal substrates for improved control and use of BCIs are not entirely understood (Soekadar et al., 2015a).

A large remaining area of study is to demonstrate specific features of how these internally interfaced devices affect changes in network connectivity. For example, a method to measure the putative changes in anatomical connectivity between two artificially linked areas would be to look at the number of projections from one area to the other in animals with and without the device post mortem; this provides a statistical means for comparison between groups, but is limited in the description of functional connectivity that may take place. Alternatively, means of visualizing connections in the brain such as diffusion tensor imaging has been used in rats *in vivo* (Laitinen et al., 2015), and could be employed for such a within-subject comparison study; however, it can be cumbersome to use such methods to map animals pre- and post- implantation. Additionally, implanted devices can obscure the accuracy of such data collection methods.

Rather than tracking changes in anatomical connectivity, it may be easier to track changes in effective connectivity directly using electrophysiological means. It is common practice to use methods such as finding the cross correlation over a sliding window to determine the average cross correlation for spike train firing in two areas in *in vitro* studies (Perkel et al., 1967). This method has also been used *in vivo* (Murphy et al., 1985), and has recently been used in conjunction with delayed mutual information to provide insight to the direction of connections as well as the specific patterns of connectivity of individual neurons (Taghva et al., 2012; Endo et al., 2015). Using statistical analyses such as cross correlation and time delayed mutual information may allow for the quantification of these effective changes over time in BCI models.

Eventually, these methods could pinpoint the time scale over which permanent changes take place, or help to identify other parameters necessary for the optimization of such devices. For example, for the closed-loop system used by Guggenmos et al. (2013) to be generalized to multiple areas of the brain, it will be necessary to test whether the delay between trigger and stimulation is a general property of ADS, or if other factors such as distance and intrinsic connectivity between areas plays a role as well. In order to test different delay times and how well they change the effective connectivity between areas, having a good metric to describe and compare changes will be critical.

## Conclusion

An ideal high fidelity BCI would both sample and allow stimulation of precise neural features non-invasively. In reality, such a combination is unlikely. Nonetheless, current work across several types of BCIs provides promising results for the clinical applicability of these technologies. Despite the positive outlook for the future of BCIs, several challenges remain before high fidelity recording and stimulating devices are made available for common clinical use. For MEA recordings, two major challenges remain. The first is to improve the patency of chronically implanted microelectrodes so that they can continue to be used for recordings for the duration of the patient’s lifespan. The second challenge is to find reliable recording sites and decoding algorithms that do not need to be recalibrated on a daily or weekly basis, and adaptive decoders that would allow for automatic recalibration as patients learn to use implanted BCIs more efficiently.

For ECoG and EEG, the challenge is less from a materials perspective, and more from a computational perspective. The primary goal remains similar to MEA-based BCIs: it is most important to find regions from which task-related information can be reliably decoded and translated into repeatable intent. It may first be necessary to find a means to identify reliable neural substrates for BCI learning using MEAs, then demonstrate that the activity patterns of these substrates can be reliably decoded using less invasive measures. Emerging methods incorporating structural and metabolic information into current source estimates may provide the additional information necessary to increase decoding accuracy (Aihara et al., 2012). Additionally, as frequency-domain based decoders improve in accuracy, it will be important to continue to incorporate signals with greater numbers of independent features into BCI decoders in order to improve the ease of adaptation for implanted patients. In ECoG, this could potentially be improved by optimizing location and spacing.

In terms of decoded output, goal-tuned single units in MEA-based BCIs have shown great promise for decoding intent in complex movements. Meanwhile, work involving less-invasive approaches such as ECoG and EEG continues to improve in decoding accuracy. The future combination of these lines of work will be critical for progress towards increased clinical use of neural prosthetics. In order to demonstrate the complete neural electrophysiological basis for learned BCI behavior, elements from all types of recording paradigms may be necessary. Such an understanding may lead to new therapeutic targets for BCI devices.

As non-invasive electrical stimulation becomes a more realistic possibility in restorative devices that use overt, extrinsic goals for patient rehabilitation, combination stimulation approaches may increase the utility and effectiveness of BCIs. Devices controlling intrinsic operations, which offer a more subtle form of closed-loop stimulation, face a different set of challenges going forward. The primary challenge will be to find a way to generalize their use to many parts of the CNS. Whether that is finding the optimal delay for ADS between two areas, or finding the right recorded or computed pattern of neural stimulation to recreate lost functionality, the challenges facing internally interfaced devices are also numerous.

Thus, the current state of progress in implementing a high fidelity BCI depends on the type of device. Restorative closed-loop devices for rehabilitation therapy have already demonstrated some clinical effect in paretic patients (Ramos-Murguialday et al., 2013), but are limited to treatment of patient populations that retain spared neural pathways following injury. Devices that control extrinsic operations have attained clinical use in the sense that they have been implemented in limited human trials (Hochberg et al., 2012; Aflalo et al., 2015). However, the practicality of such devices for widespread use remains questionable until such time that costs are reduced and devices made more widely available. In addition, decoders must be made generalizable and receive more accurate input from a higher density of sources. Completely intrinsic closed-loop devices offer tantalizing possibilities due to the possibility of not only use in motor recovery (Guggenmos et al., 2013; McPherson et al., 2015), but potentially cognitive therapy as well (Berger et al., 2012). Still, many important questions remain unanswered about these devices. Can they show reliability in animal models at a large scale? How long must such a therapeutic device remain in effect before clinical results are demonstrated? Thus, each type of device has its potential benefits and drawbacks, but importantly, an abundance of paths remain toward a future where BCIs are commonplace in a variety of clinical settings.

## Funding

This work was made possible by a generous gift from the Robert D. Deffenbaugh Foundation as well as by support from Department of Defense Congressionally Directed Medical Research Program (grant no. PT090167P1), National Institute of Neurological Disorders and Stroke/NIH (grant no. R37 NS30853), and the Kansas Training Program in Neurological and Rehabilitation Sciences (grant no. NIH 5 T32 HD 57850).

## Conflict of Interest Statement

The authors declare that the research was conducted in the absence of any commercial or financial relationships that could be construed as a potential conflict of interest. Randolph J. Nudo is co-founder and interim CEO of NeuraLink Technologies, LLC.
